# Rhythm-centred music making in community living elderly: a randomized pilot study

**DOI:** 10.1186/s12906-017-1825-x

**Published:** 2017-06-14

**Authors:** Angela Frances Yap, Yu Heng Kwan, Chuen Seng Tan, Syed Ibrahim, Seng Bin Ang

**Affiliations:** 1 Duke-NUS Medical School, 8 College Road, Singapore, 169857 Singapore; 2 Saw Swee Hock School of Public Health, National University of Singapore, 21 Lower Kent Ridge Road, Singapore, 119077 Singapore; 3 Department of Pharmacy, Khoo Teck Puat Hospital, Singapore, Singapore; 4 Singapore Heart Foundation, Singapore, Singapore; 5 OneHeartBeat Percussions, 69A Frankel Avenue, Singapore, 458197 Singapore; 6 Family Medicine Unit, KK Women’s and Children’s Hospital, 100 Bukit Timah Road, Singapore, 229899 Singapore

**Keywords:** Geriatrics, Music, Pilot projects, Quality of life

## Abstract

**Background:**

Quality of life has become an important aspect in the measurement of the health of an individual as the population ages. Rhythm-centred music making (RMM) has been shown to improve physical, psychological and social health. The purpose of this study was to explore the effects of RMM on quality of life, depressive mood, sleep quality and social isolation in the elderly.

**Methods:**

A randomised controlled trial with cross over was conducted. 54 participants were recruited with 27 participants in each arm. In phase 1, group A underwent the intervention with group B as the control. In phase 2, group B underwent the intervention with group A as the control. The intervention involved 10 weekly RMM sessions. Patient related outcome data which included European Quality of Life-5 Dimensions (EQ5D), Geriatric Depression Scale (GDS), Pittsburg Sleep Quality Index (PSQI) and Lubben Social Network Scale (LSNS) scores were collected before the intervention, at 11th and at the 22nd week.

**Results:**

A total of 31 participants were analyzed at the end of the study. The mean age was 74.65 ± 6.40 years. In analysing the change in patient related outcome variables as a continuous measure, participation in RMM resulted in a non-significant reduction in EQ5D by 0.004 (95% CI: -0.097,0.105), GDS score by 0.479 (95% CI:-0.329,1.287), PSQI score by 0.929 (95% CI:- 0.523,2.381) and an improvement in LSNS by 1.125 (95% CI:-2.381,0.523). In binary analysis, participation in RMM resulted in a 37% (OR = 1.370, 95% CI: 0.355,5.290), 55.3% (OR = 1.553, 95% CI: 0.438,5.501), 124.1% (OR = 2.241, 95% CI = 0.677,7.419) and 14.5% (OR = 1.145, 95% CI = 0.331,3.963) non-significant increase in odds of improvement in EQ5D, GDS, PSQI and LSNS scores respectively.

**Conclusion:**

Participation in RMM did not show any statistically significant difference in the quality of life of the participants. It is however, an interesting alternative tool to use in the field of integrative medicine. Moving forward, a larger study could be performed to investigate the effects of RMM on the elderly with an inclusion of a qualitative component to evaluate effects of RMM that were not captured by quantitative indicators.

**Trial registration:**

This trial was retrospectively registered. This trial was registered in the Australian New Zealand Clinical Trials Registry under trial number ACTRN12616001281482 on 12 September 2016.

**Electronic supplementary material:**

The online version of this article (doi:10.1186/s12906-017-1825-x) contains supplementary material, which is available to authorized users.

## Introduction

### Background

The silver tsunami has hit Asia with the population aged 65 and above projected to increase by more than 300% from 207 million in 2000 to 857 million in 2050 [1]. Since 1949, the World Health Organization has defined health as “a state of complete physical, mental, and social well-being and not merely an absence of disease and infirmity”. With people living longer than before, the concept of quality of life, which comprises of objective descriptors and subjective evaluations of physical, material, social, and emotional wellbeing together with the extent of personal development and purposeful activity [2], has become an important aspect in the measurement of the health of an individual and it has been recognized as an important health outcome [3]. The self-assessed health status is also a powerful predictor of morbidity and mortality [4, 5]. Integrative medicine is an emerging field [6] that has been defined as “patient-centred, healing oriented, and embracing conventional and complementary therapies” with a focus on the person as a whole, including one’s lifestyle, not just the physical body itself. It addresses the importance of nonphysical influences like emotional, spiritual and social health on physical health and disease [7] and it has been shown to be helpful in improving care for patients [8].

Depression and sleep duration has been found to have effects on health-related quality of life [9, 10]. Depression is a common mood disorder among the elderly [11, 12] which has been listed as one of the top ten causes of disability burden (in years of life lost to disability) in Singapore [13]. It is known that sleep and its characteristics have various effects on the physiology of the human body [14] and that having adequate sleep and good sleep quality is an important part in maintaining a healthy lifestyle [15].

Rhythm-centred music making (RMM), defined as the active playing of drums and various other percussion instruments, has been found to have benefits in areas of emotional, psychological and social outcomes [16, 17] with improvements in mood, reduction in anxiety, stress relief and relaxation [16, 18, 19]. The notion of group drumming has been adopted by numerous communities to promote wellness, teambuilding and sense of empowerment [20–22]. In group drumming, participants gather around in a circle each with their own instrument [23]. The session will be led by a facilitator who creates an environment that encourages participants to communicate with one another, to express their emotions and to reduce their stress and tension within through the use of various percussion instruments [24]. The drum circle connects different individuals, encouraging a sense of community [25]. This therapeutic experience through music making empowers the individual and allows them to take the intervention into their own hands. On top of the effects on emotional, psychological and social outcomes, it has also been shown by one study that drumming could lower blood pressure post-intervention [19], postulating the possibility of it bringing about improvements in the physical health of the elderly.

### Objectives

The active playing of rhythmic music using various percussion instruments, including drumming, is a type of physical activity [19] whereby the effects on the elderly has yet to be adequately explored. The purpose of this study was to explore the benefits that RMM has on the quality of life of the elderly. The effects of RMM on depressive symptoms, sleep quality and social isolation in the elderly were also studied as secondary outcomes. With the results from this study, RMM could be used as a possible tool for clinicians in the field of Integrative Medicine. A wider scale of implementation of RMM as a recreational activity could also improve the health outcomes of the elderly living in the community.

## Methods

### Trial design

This was a randomised controlled trial with cross over consisting of 2 phases and a planned allocation ratio of 1:1. Before the start of Phase 1, participants from both Group A and Group B had to complete their first set of questionnaires. In Phase 1 of the study, participants in Group A underwent the intervention while participants in Group B served as the control.

After 11 weeks, a second set of questionnaires were administered to participants from both groups. In Phase 2 of the study, participants in group A served as a control group for group B while participants in group B underwent the intervention. At the end of 11 weeks, the final set of questionnaires were administered to participants from both groups.

This study was approved by the SingHealth Centralised Institutional Review Board. Informed consent was obtained from the participants prior to participation in the study. There were no changes to methods after trial commencement.

### Participants

A total of 54 participants who met the following criteria were recruited from the community by the co-investigator YH between 1 November 2015 to 9 January 2016: (1) Aged 65 years and older; (2) Understands English or Mandarin. Exclusion criteria included: Individuals on palliative care and individuals who were bed-bound. Sociodemographic, clinical and patient related outcome data were collected via interviewer-administered questionnaires in the participant’s home.

### Intervention

Participants participated in RMM sessions of 1 h per session, under the Rhythm Wellness Programme by OneHeartBeat, held once a week for a total of 10 sessions over 11 weeks where a break was scheduled in the 4th week to factor in a public holiday within the intervention period of one of the groups. All RMM sessions were facilitated by 3 experienced instructors. The instruments used include the conga, cowbell, Djembe, Ashiko Tan-tans, Dunun, Shakers, and Wood Blocks in all the sessions.

Each session was carried out in a circle, with the participant seated comfortably and the drum or percussion instrument within reach in front of them. During each session, the instructors facilitated free play, and encouraged the participants to express themselves and interact with each other through the active playing of the instruments.

### Outcomes

Sociodemographic data included age, gender, race, marital status, number of children, type of housing and the number of people the participants are living with. Participants’ social support and social isolation were assessed using the Lubben Social Network Scale (LSNS) as well as questions pertaining to their attendance at social activities like residents/community development committee or neighbourhood events and places of worships. Clinical variables included self-reported co-morbidities, functional status, whether they were under palliative care, their hearing and vision as well as participation in physical exercise. The sociodemographic data of the participants are shown in Table 1.

**Table 1:** Participants’ socio-demographic characteristics at baseline

Variable	Total (*N* = 31)	Group A (*N* = 16)	Group B (*N* = 15)	*p*-value
Age (years)	74.65 ± 6.40	74.38 ± 6.84	74.93 ± 6.11	0.813
Gender				0.742
Female	29 (94%)	15 (94%)	14 (93%)	
Ethnic group				
Chinese	31 (100%)	16 (100%)	15 (100%)	
Highest Education Level				0.550
Primary	21 (68%)	10 (62%)	11 (73%)	
Secondary	5 (16%)	4 (25%)	1 (7%)	
Tertiary	5 (16%)	2 (13%)	3 (20%)	
Marital Status				0.674
Single	3 (10%)	2 (13%)	1 (6%)	
Married	16 (52%)	5 (31%)	7 (47%)	
Others	12 (38%)	9 (56%)	7 (47%)	
Housing Type				0.484
Private	1 (3%)	0 (0%)	1 (7%)	
HDB	30 (97%)	16 (100%)	14 (93%)	
Number of bedrooms				0.084
2 rooms or less	9 (29%)	2 (13%)	7 (47%)	
3 rooms	16 (52%)	11 (69%)	5 (33%)	
4 rooms or more	6 (19%)	3 (18%)	3 (20%)	
Number of children				0.664
0 children	4 (13%)	2 (13%)	2 (13%)	
1 child	6 (19%)	2 (13%)	4 (27%)	
2 children	8 (26%)	4 (25%)	4 (27%)	
3 or more children	13 (42%)	8 (29%)	5 (33%)	
Number of people stay in the same house				0.774
0	9 (29%)	4 (25%)	5 (33%)	
1	8 (26%)	3 (19%)	5 (33%)	
2	9 (29%)	5 (31%)	4 (27%)	
3 or more	5 (16%)	4 (25%)	1 (7%)	
Attendance at social activities				0.654
At least once a week	26 (84%)	14 (88%)	12 (80%)	
Less than once a week	5 (16%)	2 (12%)	3 (20%)	
Physical activities				1.000
At least once a week	28 (90%)	14 (88%)	14 (93%)	
Less than once a week	3 (10%)	2 (12%)	1 (7%)	
ADL				0.654
Independent	26 (84%)	14 (88%)	12 (80%)	
Require assistance	5 (16%)	2 (12%)	3 (20%)	
Number of co-morbidities				1.000
0–2	8 (26%)	5 (31%)	3 (20%)	
3–4	17 (55%)	8 (50%)	9 (60%)	
5 or more	6 (19%)	3 (19%)	3 (20%)	

Data was analysed as per intention to treat protocol. Data is presented as per Mean ± Standard Deviation for age and for the other variables, the number of participants as well as the percentage were reflected

*Abbreviations*: *N* Number, *HDB* Housing Development Board, *ADL* Activities of Daily Living

Patient related outcome data included European Quality of Life-5 Dimensions (EQ5D), Geriatric Depression Scale (GDS), Lubben Social Network Scale (LSNS) and Pittsburgh Sleep Quality Index (PSQI) scores. EQ5D, GDS, LSNS and PSQI were collected at 3 different time points - before the intervention and at the 11th and 22th week. There were no changes to trial outcome measures after the trial commenced.

The EQ-5D form is a measure of health status from the EuroQol Group primarily designed for self-completion by respondents. The score ranges from 0 (death) to 1 (full health) and includes 5 dimensions namely “mobility”, “self-care”, “usual activities”, “pain/discomfort”, “anxiety/depression” [26]. It is validated locally by Luo N [27, 28].

The GDS is a tool for the elderly populations consisting of 15 items which evaluates the level of depressive symptoms of an individual over the past week. A cut-off score of more than 5 out of the maximum 15 indicates the presence of clinically significant depressive symptoms. This scale was developed by Yesa-vage et al. [29] and validated locally by Nyunt, M. S. [30].

PSQI is a self-reported measure of sleep quality, consisting of a 19 item scale grouped into 7 equally weighted component scores: Subjective Sleep Quality, Sleep Latency, Sleep Duration, Habitual Sleep Efficiency, Sleep Disturbances, Use of Sleeping Medication and Daytime Dysfunction. The subscale scores range from 0 to 3 and the global score range from 0 to 21 with poorer sleep quality indicated by a higher global score [31, 32].

LSNS is a self-reported measure designed to gauge social isolation in older adults. It measures the frequency, size and closeness of contacts for the respondent’s social network by assessing their perceived level of support received from their friends and families. LSNS-6 is the abbreviated version with 3 items on the friends scale and 3 items on the family scale. It is used mainly to identify people that may require assistance or would benefit from linking to community programs/services [33]. The score range from 0 to 30, with a higher score reflecting more social engagement. Individuals with a score of less than 12 were identified as socially isolated.

### Sample size calculation

This was based on our primary objective which was to understand the impact of the intervention on the quality of life of the participants. The total sample size required was 30 with 15 in each arm. We used 0.074 as the minimally important clinical difference for EQ5D with a standard deviation of 0.066 [34]. We based the our calculations on the following formula, n = (Z_α/2_ + Z_β_)^2^ *2*σ^2^/d^2^, with type I error of 0.05 and power of 0.8, taking into account a dropout rate of approximately 15%.

### Randomization

A list of participants was generated by co-investigator AY and sequential numbers were assigned to each participant. Participants were randomly allocated into Group A or B using a computer random number generator. This was done by the co-investigator AY. The participants and co-investigator YH were not aware of the treatment allocation until after they were enrolled in the study. The randomization list was made available to co-investigator YH on the day of data collection and participants were notified of their group assignment verbally by YH. There was no blinding done.

### Statistical methods

Data from each of the experimental phases, Phase 1 and Phase 2 were analysed using STATA SE14.0. Within group differences for sociodemographic variables were analysed with t-test for continuous variables and Fisher exact test for categorical variables. Descriptive statistics were presented in mean ± standard deviation for continuous variables and number of participants with the percentages for categorical variables. Within group differences were analysed with Wilcoxon signed rank test and between group differences were analysed with Mann-Whitney U tests. The data was analysed based on per-protocol analysis whereby only participants who have completed the 3 data collection forms were analysed.

EQ5D, PSQI, GDS and LSNS were categorized into binary outcomes based on absolute improvements to analyze the effect of RMM on the improvements of the various outcome measures. For the continuous and binary characterization of change for each patient outcome variables, the generalized linear models using generalized estimating equation (GEE) was performed on the data to account for the correlation between repeated changes in scores. The linear predictor included the intervention indicator, order indictor, phase and the baseline patient outcome variables before the start of each phase. *P*-values less than 0.05 were reported as significant.

## Results

### Participant flow

**Fig. 1:**
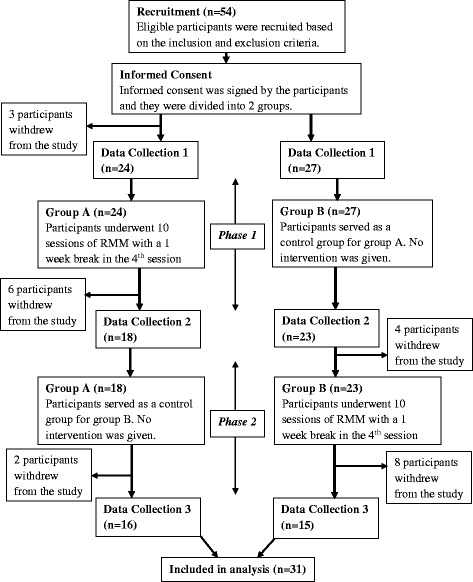
Study flow chart depicting dropouts at each time point of the study, the different data collection time points as well as the cross over design

As shown in Fig. 1, 3 participants from Group A withdrew from the start of the study due to time commitments with 24 participants left in Group A and 27 participants left in Group B. During Phase 1 of the study, a total of 6 participants from Group A and 4 participants from Group B withdrew from the study. For Group A, 1 participant dropped out because of the travelling distance to the sessions, 1 participant was un-contactable while the other 4 participants dropped out because of family and other commitments. For Group B, 2 participants from withdrew because of medical reasons and 2 participants were un-contactable. During Phase 2 of the study, a total of 2 participants in Group A and 8 participants in Group B withdrew from the study. For Group A, 1 participant passed away while the other participant was un-contactable. For Group B, 1 participant from withdrew because of social issues, 3 participants withdrew because of travelling distance while the other 4 withdrew because of time commitments. A sensitivity analysis was performed on the sociodemographic of the dropouts compared to the participants that remained and only the gender was found to be statistically significant (*p* = 0.045) as shown in an additional file [see Additional file 1].

### Recruitment

Participants were recruited from December 2015 to January 2016 and followed up till June 2016. The trial was completed as planned in June 2016.

### Baseline data

Table 1 provides the summary statistics of the sociodemographic variables for Group A and B, and there were no significant differences in sociodemographic characteristics between the two groups. The overall mean age of the study participants was 74.65 ± 6.40 and 94% of the participants were female.

### Numbers analysed

A total of 31 participants were included in the analysis, 16 from Group A and 15 from Group.

### Outcomes and estimation

Pre-intervention and Post-intervention values

In Phase 1 of the study, Group A underwent 10 sessions of RMM. As shown in Table 2, there was a non-significant decrease in median GDS scores from 3.50 (1.00, 6.00) before the intervention to 2.00 (1.00, 3.00) after the intervention and a non-significant decrease in median LSNS scores from 18.50 (7.50, 23.00) before the intervention to 12.50 (8.50, 18.50) after the intervention. The median EQ5D scores showed a non-significant improvement from 0.81 (0.67, 0.94) pre-intervention to 0.94 (0.72, 1.00) post-intervention. The median PSQI scores remained unchanged. In Phase 2 of the study, Group B underwent 10 sessions of RMM while Group A served as a control. Showing a similar trend, there was a non-significant decrease in median GDS scores from 4.00 (3.00, 6.00) before the intervention to 2.00 (1.00, 5.00) after the intervention and a non-significant decrease in median LSNS scores from 12.00 (6.00, 14.00) before the intervention to 11.00 (6.00, 17.00) after the intervention. The median EQ5D scores showed a non-significant improvement from 0.60 (0.35, 0.86) pre-intervention to 0.63 (0.39, 0.74) post-intervention. The median PSQI scores remained unchanged. It was also noted that the median GDS scores of Group A continued to show a non-significant reduction from 2.00 (1.00, 3.00) to 1.00 (1.00, 3.50).

**Table 2:** Median values of EQ5D, GDS, PSQI and LSNS at the different data collection points

	Group A			Group B			*p*-value
	*T1*	*T2*	*T3*	*T1*	*T2*	*T3*	
EQ5D	0.81 (0.67,0.94)	0.94 (0.72,1.00)	0.87 (0.69,0.88)	0.63 (0.28,0.74)	0.60 (0.35,0.86)	0.63 (0.39,0.74)	0.022
GDS	3.50 (1.00,6.00)	2.00 (1.00,3.00)	1.00 (1.00,3.50)	6.00 (3.00,8.00)	4.00 (3.00,6.00)	2.00 (1.00,5.00)	0.124
PSQI	4.00 (3.00,7.50)	4.00 (4.00,7.00)	5.00 (4.00,8.00)	8.00 (6.00,10.00)	7.00 (5.00,10.00)	7.00 (3.00,10.00)	0.068
LSNS	18.50 (7.50,23.00)	12.50 (8.50,18.50)	12.00 (8.00,16.00)	10.00 (4.00,14.00)	12.00 (6.00,14.00)	11.00 (6.00,17.00)	0.049

Values are in Median (25th percentile, 75th percentile). T1,T2,T3 represents 1st, 2nd and 3rd data collection time points respectively

*Abbreviations*: *EQ5D* European Quality of Life-5 Dimensions, *GDS* Geriatric Depression Scale, *PSQI* Pittsburg Sleep Quality Index, *LSNS* Lubben Social Network Scale

*p*-value is obtained from Mann-Whitney U test between Group A and Group B at T1 on patient related outcome variables

### Effects of RMM on patient outcome variables

In studying the effects of RMM on the change of EQ5D, GDS, PSQI and LSNS as a continuous measure, a linear regression model using GEE was performed where the outcome is the change in scores while taking into account phase, order and baseline EQ5D, GDS, PSQI and LSNS respectively before the start of each phase, and the correlation across repeated measurements. As shown in Table 3, it is noted that participation in RMM resulted in a non-significant reduction in EQ5D by 0.004 (95% CI: -0.097, 0.105), GDS score by 0.479 (95% CI:-0.329, 1.287), PSQI score by 0.929 (95% CI:-0.523, 2.381) and an improvement in LSNS by 1.125 (95% CI:-1.134, 3.384).

**Table 3:** Effects of RMM on EQ5D, GDS, PSQI and LSNS

	Continuous			Binary		
	Coefficient	(95% CI)	*p*-value	OR	(95% CI)	*p*-value
EQ5D	−0.004	(−0.105,0.097)	0.935	1.370	(0.355,5.290)	0.647
GDS	−0.479	(−1.287,0.329)	0.245	1.553	(0.438,5.501)	0.496
PSQI	−0.929	(−2.381,0.523)	0.210	2.241	(0.677,7.419)	0.186
LSNS	1.125	(−1.134,3.384)	0.329	1.145	(0.331,3.963)	0.831

Values shown have been adjusted for order and period effect

*Abbreviations*: *OR* Odds Ratio, *CI* Confidence Interval, *EQ5D* European Quality of Life-5 Dimensions, *GDS* Geriatric Depression Scale, *PSQI* Pittsburg Sleep Quality Index, *LSNS* Lubben Social Network Scale, *ADL* Activities of Daily Living

In classifying the patient outcome variables into improvements vs no improvement, a logistic regression model using GEE was performed while taking into account phase, order and baseline patient outcome variables before the start of each phase, and the correlation across repeated measurements. It is noted that participation in RMM resulted in a 37% non-significant increase in odds of improvement in EQ5D scores (OR = 1.370, 95% CI: 0.355,5.290), 55.3% non-significant increase in odds of improvement in GDS scores (OR = 1.553, 95% CI: 0.438,5.501), 124.1% non-significant increase in odds of improvement in PSQI scores (OR = 2.241, 95% CI = 0.677,7.419) and a 14.5% non-significant increase in odds of improvement in LSNS scores (OR = 1.145, 95% CI = 0.331,3.963) when compared with no participation in RMM.

### Ancillary analysis

Ancillary analysis was not performed.

### Harms

The co-investigators YH and AY enquired about any adverse events by asking the participants for feedback, allowing them to raise issues at the end of each session, finding out how they felt about the sessions and whether there were any issues or discomfort they might have experienced in between each session. There were no adverse issues raised by the participants.

## Discussion

### Interpretation

This is the first study performed evaluating the effects of RMM on the quality of life and sleep quality of individuals providing the basis for future studies to explore these areas. The strengths of the study include a crossover study design that provided a control for each intervention arm through randomization and the generalized linear models using GEE to account for phase, order and baseline patient outcome effects.

It is noted from Table 2 that the baseline patient related outcome variables for both groups were significantly different when comparing EQ5D and LSNS. Thus, an adjusted analysis was performed with the results reflected in Table 3.

The effects of RMM on EQ5D scores as a primary outcome showed a non-significant decrease in quality of life in the continuous analysis and a non-significant increase in odds of improvement in EQ5D scores. In studying the effects of RMM on GDS, PSQI and LSNS scores as secondary outcome measures after correction for baseline patient related outcome variables, results showed a general trend of increasing the probability of improvement in depressive mood, sleep quality and social isolation with participation in RMM even though it was not statistically significant for both continuous and binary analysis. The effects of RMM on the psychological health of participants with improvements in anxiety [35], mood [36], stress, anger and self-esteem [37] has been shown in various other studies as well [38].

Even though the scores for EQ5D as the primary outcome measure were not significantly different post-intervention, the RMM sessions were well-received by the majority of the participants of the intervention group. RMM could be a new activity adopted by various communities and nursing homes to engage the elderly as a form of recreational activity with benefits on the health of an individual. It could also be a possible non-pharmacological therapy in the field of Integrative Medicine where the focus is not only on the physical health but also on treating the patient whole including their lifestyle as well.

### Generalizability

The participants of this study were from a population of community living elderly in Singapore. Hence, the results of the study could be applicable to the elderly population locally.

### Limitations

Results from the study showed possible improvements in depressive mood, sleep quality and social isolation in individuals but due to limited statistical power from a small sample size as this is a pilot study, the changes were not statistically significant. Also, as it is difficult to comprehensively quantify the benefits of RMM on the individual participants, a qualitative study could be conducted in the future to objectively study the effects of RMM that were not captured by the patient-related outcome measures in this study. However, this quantitative study provides the ground work for future exploration of the impact of this intervention on quality of life, sleep quality, depression and social isolation. With regards to future studies conducted on RMM, if a cross over study design was to be chosen, a suitable duration for a washout period could be incorporated to take into account a carryover effect from the intervention. A randomized controlled trial design without crossover could be considered as it is difficult to quantify a sufficient washout period and the intervention may result in a learned effect unlike drugs, resulting in an incomplete washout.

## Conclusion

Participation in RMM did not show any statistically significant difference in the quality of life of participants. It is however, an interesting alternative tool to use in the field of integrative medicine and moving outside the field of medicine, it could be a new form of recreational activity adopted by the elderly in the community as well as in nursing homes. Moving forward, a larger study could be performed to investigate the effects of RMM on the elderly with an inclusion of a qualitative component to evaluate effects that were not captured by quantitative indicators.

## Additional file

Additional file 1: Socio-demographic characteristics of participants who dropped out and participants who remained. (DOCX 14 kb)

## Abbreviations

ADL: Activities of Daily Living
CI: Confidence Interval
EQ5D: European Quality of Life-5 Dimensions
GDS: Geriatric Depression Scale
GEE: Generalized estimating equation
HDB: Housing Development Board
LSNS: Lubben Social Network Scale
N: Number
OR: Odds Ratio
PSQI: Pittsburg Sleep Quality Index
RMM: Rhythm-centred Music Making
T1: First data collection time point
T2: Second data collection time point
T3: Third data collection time point
