# Supplementary material for: Rhythm-centred music making in community living elderly: a randomized pilot study
Source: BMC Complement Altern Med. 2017 Jun 14;17:311. doi: 10.1186/s12906-017-1825-x (PMC5470187; doi:10.1186/s12906-017-1825-x)
Supplement: Additional file 1: — Socio-demographic characteristics of participants who dropped out and participants who remained. (DOCX 14 kb) [file 12906_2017_1825_MOESM1_ESM.docx]

| **Annex 1: Socio-demographic characteristics of participants who dropped out and participants who remained** | | | |
| --- | --- | --- | --- |
| **Variable** | **Participants who dropped out (N=20)** | **Remaining Group of participants (N=31)** | **p-value** |
| **Age** (years) | 72.90 ± 7.09 | 74.65± 6.40 | 0.648 |
| **Gender**  Female | 14 (70%) | 29 (94%) | 0.045 |
| **Ethnic group**  Chinese | 18 (90%) | 31 (100%) | 0.149 |
| **Highest Education Level** |  |  | 0.757 |
| Primary | 13 (65%) | 21 (68%) |  |
| Secondary | 5 (25%) | 5 (16%) |  |
| Tertiary | 2 (10%) | 5 (16%) |  |
| **Marital Status** |  |  | 0.650 |
| Single | 3 (15%) | 3 (10%) |  |
| Married | 12 (60%) | 16 (52%) |  |
| Others | 5 (25%) | 12 (38%) |  |
| **Housing Type** |  |  | 0.287 |
| Private | 3 (15%) | 1 (3%) |  |
| HDB | 17 (85%) | 30 (97%) |  |
| **Number of bedrooms** |  |  | 0.864 |
| 2 rooms or less | 7 (35%) | 9 (29%) |  |
| 3 rooms | 10 (50%) | 16 (52%) |  |
| 4 rooms or more | 3 (15%) | 6 (19%) |  |
| **Number of children** |  |  | 0.393 |
| 0 children | 5 (25%) | 2 (13%) |  |
| 1 child | 0 (0%) | 4 (27%) |  |
| 2 children | 6 (30%) | 4 (27%) |  |
| 3 or more children | 9 (45%) | 5 (33%) |  |
| **Number of people stay in the same house** |  |  | 0.792 |
| 0 | 6 (30%) | 4 (13%) |  |
| 1 | 3 (15%) | 6 (19%) |  |
| 2 | 7 (35%) | 8 (26%) |  |
| 3 or more | 4 (20%) | 13 (42%) |  |
| **Attendance at social activities** |  |  | 0.178 |
| At least once a week | 13 (65%) | 26 (84%) |  |
| Less than once a week | 7 (35%) | 5 (16%) |  |
| **Physical activities** |  |  | 0.411 |
| At least once a week | 16 (80%) | 28 (90%) |  |
| Less than once a week | 4 (20%) | 3 (10%) |  |
| **ADL** |  |  | 0.384 |
| Independent | 19 (95%) | 26 (84%) |  |
| Require assistance | 1 (5%) | 5 (16%) |  |
| **Number of co-morbidities** |  |  | 0.462 |
| 0-2 | 10 (50%) | 8 (26%) |  |
| 3-4 | 6 (30%) | 17 (55%) |  |
| 5 or more | 4 (20%) | 6 (19%) |  |
